# Comparison of ropivacaine plus sufentanil and ropivacaine plus dexmedetomidine for labor epidural analgesia: A randomized controlled trial protocol

**DOI:** 10.1097/MD.0000000000022113

**Published:** 2020-09-04

**Authors:** Gang Chen, Maowei Gong, Yi Liu

**Affiliations:** Department of Anesthesia, The First Medical Center of Chinese PLA General Hospital, Beijing 100853, China.

**Keywords:** dexmedetomidine, epidural analgesia, labor pain, ropivacaine, sufentanil

## Abstract

**Objective:**

Effective analgesia during delivery can not only decrease pain, but also have a significant function in ensuring the safety of baby and mother. Sufentanil is generally used opioid with ropivacaine in epidural anesthesia in labor pain management; however it can cause some adverse reaction. Dexmedetomidine is an a2-adrenoceptor agonist with high selectivity. It possesses opioid-sparing and analgesic effects and it is suitable for the long-term and short-term intraoperative sedation. The purpose of this present study is to compare the analgesic effect of ropivacaine with dexmedetomidine against ropivacaine with sufentanyl in epidural labor.

**Methods:**

This is a single center, placebo-controlled randomized trial which will be performed from May 2020 to May 2021. It was authorized via the Institutional Review Committee in the first medical center of Chinese PLA General Hospital (S2018-211-0). One hundred sixty full-term protozoa are included in this work. They are randomly divided into four groups (*n* = 40 per group): the RD1 group (with the epidural administration of 0.125% ropivacaine + dexmedetomidine of 0.5 μg/mL), and the RD2 group (with the epidural administration of 0.08% ropivacaine + dexmedetomidine 0.5 μg/mL), the RS1 group (with the epidural administration of 0.125% ropivacaine + sufentanil of 0.5 μg/mL), as well as RS2 group (with the epidural administration of 0.08% ropivacaine + sufentanil of 0.5 μg/mL). Clinical outcomes are pain score, a modified Bromage scale, the Ramsay Sedation Scale, and adverse reactions during analgesia. All the needed analyses are implemented through utilizing SPSS for Windows Version 20.0.

**Results:**

The first table shows the clinical outcomes between these four groups.

**Conclusion:**

This current work can provide a primary evidence regarding the clinical outcomes of dexmedetomidine versus sufentanil for labor epidural analgesia.

**Trial registration:**

This study protocol was registered in Research Registry (researchregistry5877).

## 1 Introduction

Labor is considered to be one of the most painful and unforgettable things in a life of woman.^[1,2]^ The physical effects of labor pain are obvious. The labor pain is a strong respiratory stimulus that leads to a significant increase in oxygen consumption and minute ventilation in the process of contractions. Stress and pain during the labor stimulates sympathetic nervous system. The increase of catecholamine is related to the reduction of the uterine blood flow, and the increase of blood pressure and cardiac output.^[3]^ Non-treatment approaches have no adverse effects on maternal and newborn health but have poor analgesic effects. Therefore, it is used in mild to moderate pain conditions. Appropriate analgesics are necessary for parturient women during labor.^[4,5]^

The gold standard for the laboring patients is nerve axis block, containing epidural block, spinal cord, or the spinal-epidural combination techniques.^[6–8]^ Other drugs associated with the axonal block or use alone include nitrous oxide, non-opioids, and opioids, distraction therapy as well as the patient-controlled analgesia.^[9,10]^ Ropivacaine is local anesthetic agent which is widely used in the surgical procedure for pain control, including general surgery, joint arthroplasties, and cardiac surgery.^[11]^ The combined use of ropivacaine with opioid, such as sufentanil, in epidural anesthesia has a significant effect on postoperative pain management, but opioid have adverse reactions such as vomiting, headache, urinary retention, and respiratory depression.^[12,13]^ Dexmedetomidine is an a2-adrenoceptor agonist with high selectivity. It possesses opioid-sparing and analgesic effects and it is suitable for the long-term and short-term intraoperative sedation.^[14]^ Published research studies have shown that the ropivacaine combined with dexmedetomidine can effectively relieve postoperative pain. The purpose of this present study is to compare the analgesic effect of ropivacaine with dexmedetomidine against ropivacaine with sufentanyl in epidural labor.

## 2 Methods

### 2.1 Study design

This is a single center, placebo-controlled randomized trial will be performed from May 2020 to May 2021, which is performed in accordance with the SPIRIT Checklist for randomized studies. It was authorized via the Institutional Review Committee in the first medical center of Chinese PLA General Hospital (S2018-211-0) and then was registered in research registry (researchregistry5877). The obstetrician will explain the details of trial, afterward, patiently answer all the questions from primigravidae. The primigravidae is then presented with the written information of our trial. Each patient received a written informed consent. Since all patients participated voluntarily, they could withdraw at any time during the trial.

### 2.2 Subjects and grouping

One hundred sixty full-term primigravidae are included in this work. In the random envelope, all participating parturients will be assigned a random number via utilizing the random number table, and the result of allocation is hidden. They are randomly divided into four groups (*n* = 40 per group): the RD1 group (with the epidural administration of 0.125% ropivacaine + dexmedetomidine of 0.5 μg/mL), and the RD2 group (with the epidural administration of 0.08% ropivacaine + dexmedetomidine 0.5 μg/mL), the RS1 group (with the epidural administration of 0.125% ropivacaine + sufentanil of 0.5 μg/mL), as well as RS2 group (with the epidural administration of 0.08% ropivacaine + sufentanil of 0.5 μg/mL). Physicians, statisticians, data collectors, and evaluators are all blinded to the allocation.

### 2.3 Inclusion of exclusion criteria

Inclusion criteria: Primigravidae aged between 20 and 35 years with a single pregnancy between 38 and 42 weeks; those without an abortion history; those with ASA I-II classification; and women with normal platelet coagulation and count function. And the exclusion criteria include parturient women with contraindications to the epidural anesthesia; the women with severe kidney, liver, lung, and heart diseases; those with serious preeclampsia or malignant neoplasms; women with a cesarean section history, and the history of urological surgery or lower abdominal surgery; long-term use of analgesics or sedatives accompanied by the systemic disease; and parturient women with a mental illness history.

### 2.4 Anesthesia method

Insert the intravenous catheter into the brachial vein followed by 5 mL/kg/h sodium lactate infusion. 3 to 4 cm toward cephalad direction, and insert epidural catheter at interspace of L3-L4. Intravenous 1.5% lidocaine with epinephrine of 3 mL is given to primigravidae. When 3 cm cervical dilation reached, the epidural anesthesia is performed. An automatic infusion pump is used for the administration of anesthetics. In this present study, infusion pump is set as follows: the loading volume is 10 mL with 8 mL/h background infusion, and the patient-controlled epidural analgesia dose is 8 mL, and the locking interval is 30 minutes. After the initial injection volume of 13 mL, the background dose of 8 mL/h is injected. If additional patient-controlled epidural analgesia is required in the process of analgesia, the background dose is readministered 1 hour after the analgesia. Patient controlled epidural analgesia would stop when the cervix is completely dilated. After delivery, the laceration or the lateral episiotomy incision is received the local anesthesia and then sutured. Afterward, remove the epidural catheter.

### 2.5 Hemodynamic parameters

Patient Monitor (Intellivue MP60, Philips) is used to measure the parameters of hemodynamics (heart rate and the diastolic and systolic blood pressure) at various stages of delivery. Hemodynamic parameters are measured by the anesthesiologists of institutes.

### 2.6 Outcome measures

Pain score was measured by nursing staff (a minimum of 3-y of experience; an unaware about treatments) of institutes by administrating visual analog scale (VAS) score at various stages of delivery. Ten: the maximum possible pain and 0: absent pain.

The improved Bromage score is utilized to grade the motor block caused by intraspinal anesthesia in the parturient women. Zero point represents no movement obstacle; 1, cannot lift the straight leg, cannot move the feet and knees; 2, cannot lift the straight leg and move the knee, and can move the feet; and 3, the movement limb is completely blocked. And Ramsay Sedation Scale is utilized to evaluate the sedation levels of patients. The score of 1, indicates anxiety, uneasiness, and irritability; 2, oriented, cooperative, and calm; 3, only responsive to the commands; 4, brisk response to stimulus; 5, sluggish response to stimulus; and 6, no response to stimulus.

Adverse reactions during analgesia are observed in these four groups, including bradycardia, respiratory depression, hypotension, nausea and vomiting, and pruritus. At the same time, urinary retention is monitored at 8 hours and 20 hours after delivery.

### 2.7 Statistical analysis

All the needed analyses are implemented through utilizing SPSS for Windows Version 20.0. All the data are represented with proper characteristics as median, mean, percentage as well as standard deviation. Independent samples *t*-test or the Mann-Whitney *U* test is utilized for the comparison between groups. Chi-square detection is utilized to compare the categorical variables among the groups. The analysis of repeated measurement of the variance is applied to analyze the repeated data. A *P* < 0.05 is regarded the significant in statistics.

## 3 Results

Table 1 will show the clinical outcomes between these four groups.

**Table 1:**
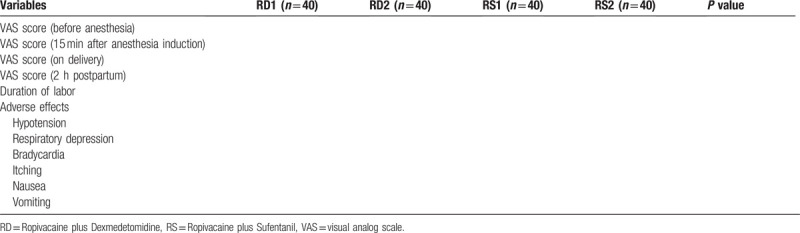
The clinical outcomes between four groups.

## 4 Discussion

The labor pain will bring great mental and physical pain to the parturient women, and meanwhile, a series of stress reactions in the process of delivery will also have a negative impact. In recent years, with the continuous development of modern medicine, labor analgesia has been extensively utilized in obstetrics.^[15,16]^ Labor analgesia (also known as painless childbirth), that is, the use of pharmacological, psychological, physical, and other related therapies to relieve the pain of maternal childbirth.^[17]^ Common approaches in this area include the non-pharmacological and pharmacological methods. Drug-induced analgesia is more popular in the clinical practice, including intravenous anesthesia, epidural anesthesia, as well as inhalation anesthesia. Effective analgesia during delivery can not only relieve pain, but also have a significant function in ensuring the safety of baby and mother.^[18]^ Sufentanil and dexmedetomidine are commonly used in epidural analgesia. However, the optimal drug and regime remain controversial. We conduct this work to compare the ropivacaine with dexmedetomidine against ropivacaine with sufentanil for the epidural analgesia in labor. The limitation of the study is that the sample size of our study is small; a multicenter randomized controlled trial is still required.

## 5 Conclusion

This current work can provide a primary evidence regarding the clinical outcomes of dexmedetomidine versus sufentanil for labor epidural analgesia.

## Author contributions

Yi Liu planned the study design. Maowei Gong reviewed the study protocol and collected data. Gang Chen finished the manuscript. All of the authors approved the article.

**Data curation:** Maowei Gong.

**Formal analysis:** Maowei Gong.

**Writing – original draft:** Gang Chen.
